# Physical activity and risk of testicular cancer: a systematic review

**DOI:** 10.1186/s12885-018-4093-3

**Published:** 2018-02-14

**Authors:** Stephanie Huang, Virginia Signal, Diana Sarfati, Caroline Shaw, James Stanley, Katherine McGlynn, Jason Gurney

**Affiliations:** 1 Cancer and Chronic Conditions (C3) Research Group, Department of Public Health, University of Otago, Wellington, New Zealand; 2 Department of Public Health, University of Otago, Wellington, New Zealand; 3 Division of Cancer Epidemiology & Genetics, National Cancer Institute, Rockville, MD USA

**Keywords:** Testicular cancer, Physical activity, Recreation, Occupation, Sport, Exercise

## Abstract

**Background:**

Physical activity has been implicated as a risk factor in the development of testicular cancer (TC), but the relationship remains controversial. This systematic review pooled available evidence regarding this association.

**Methods:**

Using Boolean search terms and following PRISMA guidelines, we examined the risk of TC across three categories of exposure: intensity (i.e. comparison of risk between those previously exposed to high, moderate and low levels of physical activity); dose-response (i.e. whether risk of TC increases or decreases with increasing exposure to physical activity); and the role of timing of physical activity (i.e. during early childhood or adolescence).

**Results:**

Thirteen studies (11 case-control studies, 2 cohort studies) were included in the review. While some studies have reported a strong protective effect of high levels of physical activity on risk of TC, others have reported either no relationship or a weak direct association; and while a dose-response relationship has been identified across several studies, this relationship has been observed in both directions. Similarly conflicting results exist in terms of individual types of activity and the lifecourse timing of the physical activity. Reasons for this inconsistency may include the absence of any association, heterogeneous assessment of physical activity, misclassification bias and difference in sample sizes.

**Conclusions:**

On balance, there is presently no strong evidence of an association between physical activity and risk of subsequent TC. This review highlights key areas for future investigation that may clarify any association between physical activity and risk of testicular cancer.

**Electronic supplementary material:**

The online version of this article (10.1186/s12885-018-4093-3) contains supplementary material, which is available to authorized users.

## Background

Testicular cancer (TC) is the most common malignancy affecting men aged 15 to 40 years, with incidence increasing steadily worldwide over the past several decades [1]. Despite many studies investigating pathogenesis of TC, the only well-established risk factors are cryptorchidism, increased adult height, and prior personal and family history of TC [2]. While current evidence suggests that TC risk is largely determined in utero [3], there is also evidence that TC risk may be influenced by postnatal factors [2]. The contribution of these factors to TC development remains poorly understood.

One of the postnatal risk factors for TC that has been investigated is physical activity. A 2010 review that pooled existing evidence regarding the association between physical activity and any type of cancer found “convincing or probable evidence” that physical activity reduces the risk of colon, breast and endometrial cancers, but “null or insufficient evidence” for reduction in TC risk [4].

The influence of different aspects of physical activity on TC development has been examined, including the frequency, intensity, duration and type of activity in recreational and occupational domains over different lifetime periods. Some studies suggest that increased recreational physical activity is associated with increased risk of TC [5, 6], while others observe decreased risk [7] or no association [8]. Inconsistency in the results is also evident in studies of occupational physical activity, with one [5] reporting increased risk and another [9] reporting decreased risk.

In light of these conflicting observations, we have conducted a systematic review of the literature in order to address the following questions: on balance, is physical activity associated with an increased (or decreased) risk of TC? If an association exists, is there a dose-response relationship? Does TC risk vary between different types of physical activity? Finally, are there certain critical periods during the lifecourse (e.g. childhood, adolescence, adulthood) at which physical activity affects the risk of TC?

## Methods

### Search strategy

This study was performed in accordance with the Preferred Reporting Items for Systematic Reviews and Meta-Analyses (PRISMA) guidelines [10].

#### Protocol and registration

We registered this review in the International Prospective Register of Systematic Reviews (PROSPERO, registration No. CRD42016051956), describing in advance the aims and methods of our investigation [11].

#### Eligibility criteria

The PICOS (**P**atient/**P**articipant, **I**ntervention, **C**omparator, **O**utcome, **S**tudy design) criteria used to construct this review are outlined in the (Additional file 1: Table S4). Abstracts included in the final analysis included studies that reported an association between the exposure (physical activity) and the outcome (testicular cancer). Studies were only included if data were provided from which summary associations (odds ratio or relative risks) and their 95% confidence limits could be calculated, or if these summary associations were provided by the authors themselves.

#### Information sources

A systematic review was conducted on 11th November 2016 for all articles published up until that time. No limits were set in terms of language used or study design during the initial abstract search. The search was conducted using Ovid Medline, Embase, Scopus and Web of Science databases. The reference lists of those studies considered eligible for inclusion (see *Study selection and data extraction*, below) were scanned for additional relevant studies.

#### Search terms

Using a Boolean approach, we searched the electronic databases for each possible combination of the following keywords (* indicates ‘explosion’ term). These are shown in Table 1. References were collected and logged in EndNote vX7.1 (Thomson Reuters, New York, U.S.A.).

**Table 1:** Search terms used during systematic review of the literature

Exposure-related keywords	Outcome-related keywords
Physical activit*	Cancer of the testi*
Exercis*	Testi*cancer
Sport*	Testi* carcinoma
Fitness*	Testi* tumour
Occupational activit*	Testi* neoplasm
Recreational activit*	Testi* germ cell tumour
Aerobic*	Seminoma
Anaerobic	Non-seminoma
	Teratoma
	Testi* Choriocarcinoma

### Study selection and data extraction

#### Screening of abstracts

Duplicate records were removed prior to abstract screening. Abstracts were screened by one reviewer (SH) to remove irrelevant studies, with a 10% random sample of these removed studies verified by a second reviewer (VS). The full text of all remaining papers was obtained and assessed by two reviewers (SH and VS) to identify those which met our inclusion criteria. Any disagreements about inclusion were resolved by referral to a third reviewer (JG). All papers that were considered relevant during the abstract screening process but ineligible for inclusion in our final analysis are listed in the Additional files, along with justification for why they were ultimately excluded (Additional file 1: Table S5).

#### Data extraction

In line with PRISMA guidelines, two reviewers (SH and VS) independently extracted meta-data for each included study. In those cases where the two reviewers disagreed with respect to a data item, the discrepancy was again resolved by the third reviewer (JG).

#### Assessment of risk of bias (individual and across studies)

While the assessment of study quality and potential for bias is an essential feature of any systematic review, there remains no gold standard measure of study quality for observational research. In the absence of such a gold standard, it has been recommended that any tools used to measure study quality should be as specific as possible to the given topic, and involve a simple checklist as opposed to a scale or score [12]. On this basis, we assessed study quality and potential for bias using the criteria outlined in the Newcastle-Ottawa Quality Assessment Scale, [13, 14] but did not determine a quality score [15]. Two reviewers (SH and VS) independently assessed study quality against these criteria, with disagreements resolved by referral to a third reviewer (JG).

## Results

### Literature search results

**Fig. 1:**
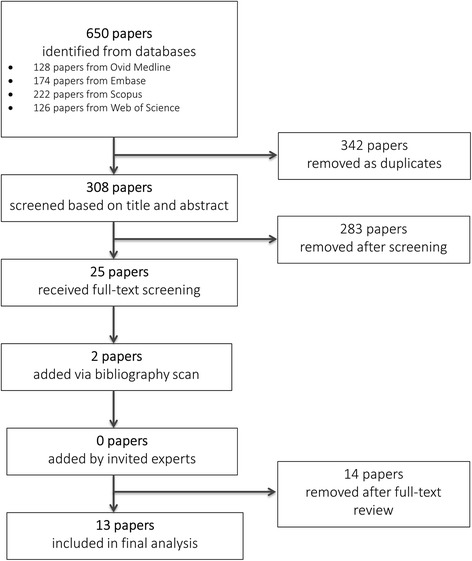
Flow chart of systematic literature search

The flow chart for the literature search strategy and results is presented in Fig. 1. Our search strategy yielded a total of 13 papers that investigated the association between physical activity and risk of TC.

### Meta-data for included studies

Meta-data for each of the 13 included manuscripts are presented in Additional file 1: Table S1, with eleven being case-control studies [5–7, 9, 16–22] and two being cohort studies [8, 23]. These 13 included manuscripts actually represented eleven distinct studies, with three of the included manuscripts drawing from the same large study. Two of the case-control studies were conducted by the UK Testicular Cancer Study Group during 1984–1987 in England and Wales, using the same study sample and method [20, 21]. These studies examined the influence of two different aspects of physical activity on TC development, with one [20] investigating the types of sports and the other [21] investigating recreational exercise duration, and so they were both included in the final analysis. Another case-control study published additional analysis based on these two studies, and since new findings were reported regarding the relationship between physical activity and TC by histology, it was also included [17].

Four of the included case-control and cohort studies were conducted in the USA, [9, 16, 22, 23] three in the UK, [17, 20, 21] three in Canada, [5–7] two in Europe [8, 19] and one in Turkey [18]. Dates of participant recruitment ranged across studies from 1970 [6] to 2006 [19, 22]. A majority of the case-control studies had moderate sample sizes, with the lowest number of cases being 128 [6] and the highest being 794 [17, 20, 21]. For the eleven case-control studies, seven obtained their cases from regional or national cancer registries [5, 7, 9, 17, 20–22], with the remainder drawing cases from hospital records and specimen banks. Eight of these case-control studies drew their controls from the community, [5, 7, 16, 17, 19–22] with the remainder drawing their controls from hospital or general practitioner records. For the two cohort studies, the earliest was among college alumni [23] and the latest among the Norwegian population [8].

### Assessment of study quality

Assessment of study quality using Newcastle-Ottawa criteria showed that, according to their criteria, there was minimal risk of bias which may have affected results (Additional file 1: Table S6 and S7). All included studies either matched cases to controls, or exposed to non-exposed for cohort studies; or adjusted in analysis for more than one confounding variables (age and other factors). There was a slight difference in non-response rate between cases and controls, with this difference ranging between 7% and 25%. The use of self-report to assess exposure status is discussed later in the manuscript, as is the risk of misclassification bias arising from the use of different measurement instruments for physical activity.

### Disparate physical activity measurements

Measurement of physical activity differed substantially across studies, both in terms of unit of measurement and lifecourse timing (Additional file 1: Table S1). Almost all included studies used self-reported assessment of physical activity collected through either written surveys or interview. However, most questionnaires did not thoroughly assess total combination of frequency, intensity, duration, type and domains of physical activity, making examination of the relationship between net physical activity and TC development difficult. For example, Brownson et al. [9] investigated occupational activity intensity by categorising job titles obtained from hospital records into high, moderate and low physical intensity occupations. Another study by Forman et al. [21] focused on recreational activity duration, based on exercise time in hours per week and time spent sitting in hours per day. The lifecourse timing of physical activity measurement across studies also ranged widely from childhood and adolescence [16, 22] to adulthood [8, 9, 18, 23], with several studies spanning multiple life periods [5–7, 17, 19–21].

Because of the disparate measurement of physical activity, meta-analyses of the data could not be performed as originally planned. However, based on the patterns of observations described in the literature – albeit made using heterogeneous measures – we have described the current state of evidence with respect to the association between physical activity and TC development below. In studies where both crude and adjusted risk estimates were presented, adjustment for confounding had little impact, so adjusted measures were used for comparison across studies.

### High vs. low levels of physical activity

**Fig. 2:**
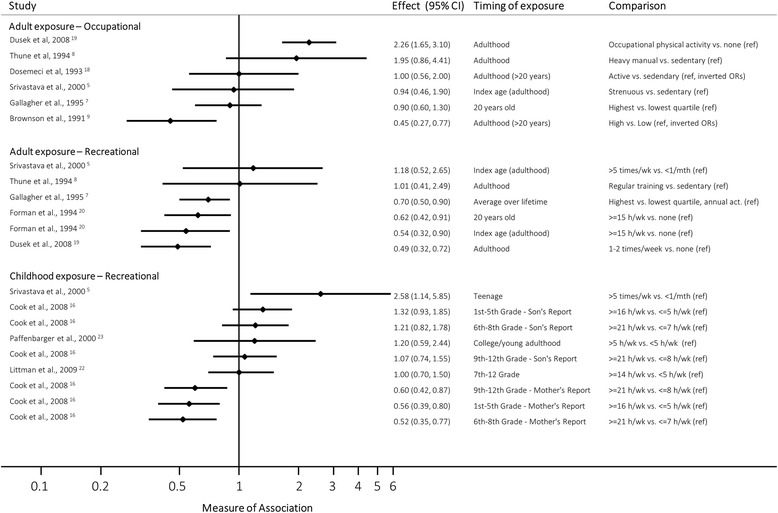
Forest plot, showing measures of association between high and low levels of recreational and occupational physical activity, by timing during lifecourse. All measures of association are odds ratios (ORs), except Thune et al. [8] and Paffenbarger et al. [23] (rate ratios, RRs). We have assumed approximate comparability between the two measures

Overall, seven studies (6 case-control, 1 cohort) described the relative risk of TC development among those exposed to high levels of physical activity compared with those with low levels (Additional file 1: Table S2), of which two examined occupational activity [9, 18], one examined recreational activity, [21] and the remaining four studies examined it in both domains [5, 7, 8, 19]. In Fig. 2, we have presented a forest plot which shows the association between high vs. low levels of recreational and occupational physical activity, by timing during lifecourse. In those cases where a study made multiple comparisons, each relevant association is presented separately along with a definition of the comparison being made and the timing of the exposure.

In terms of occupational activity, Brownson et al. [9] observed that low physical activity levels were strongly associated with increased TC risk compared to high levels (adjusted OR: 2.20, 95% CI 1.30–3.70). However, both Dusek et al. [19] and Thune et al. [8] observed essentially the opposite: i.e. an increased risk of TC with high activity levels compared to low/no activity (adjusted OR: 2.26, 95% CI 1.65–3.10; and adjusted RR: 1.95, 95% CI 0.86–4.41, respectively), while three further studies did not find evidence of any such association (Dosemeci et al. [18]: adjusted OR 1.00, 95% CI 0.50–1.80; Gallagher et al. [7]: adjusted OR 0.90, 95% CI 0.60–1.30; Srivastava et al. [5]: adjusted OR 0.94, 95% CI 0.46–1.90). Overall, the results ranged from suggesting that those doing high levels of occupational activity had less than half the odds of TC up to more than double the odds compared to those who did low levels of activity.

Similar to occupational activity, studies examining recreational activity also reported mixed results with respect to comparisons of TC risk between high and low activity levels [5, 7, 8, 19, 21]. Three studies observed a moderately strong protective association, in which those exposed to high levels of activity were at a reduced risk of TC development (Dusek et al. [19]: adjusted OR 0.49, 95% CI 0.32–0.72; Forman et al. [21]: adjusted OR 0.54, 95% CI 0.32–0.90; Gallagher et al. [7]: adjusted OR 0.70, 95% CI 0.50–0.90). However two other studies did not observe any association between physical activity and risk of TC (Srivastava et al. [5]: adjusted OR 1.18, 95% CI 0.52–2.65; Thune et al. [8]: adjusted RR 1.01, 95% CI 0.41–2.49).

Those studies that specifically looked at moderate (vs. sedentary) activity found similarly-conflicting results. For recreational activity, Gallagher et al. [7] observed that those exposed to moderate activity levels had 40% decreased risk of TC (adjusted OR: 0.60, 95% CI 0.50–0.90). However, others have not found evidence of a protective association. Srivastava et al. [5] and Thune et al. [8] compared the risk of TC between those engaged in moderate weekly activity to those who were sedentary, and observed no clear association (adjusted OR: 1.42, 95% CI 0.54–3.17 and adjusted RR:1.22, 95% CI 0.55–2.69, respectively). Forman et al. [21] similarly found no association (e.g. 3–4 h/week activity vs. 0 h/week, adjusted OR: 0.94, 95% CI 0.69–1.29).

### Dose-response relationship

Eight studies [5, 7–9, 16, 18, 21, 22] investigated the relationship between varying levels of physical activity exposure and risk of TC. Two of these studies found evidence that increasing physical activity was associated with a decreased risk of TC: Brownson et al. [9] observed that the odds of TC decreased as occupational physical demands increased (adjusted ORs: low [compared to high] 2.20, 95% CI 1.30–3.70; moderate, 1.10, 95% CI 0.80–1.70, p for trend < 0.01), while Forman et al. observed that the odds of TC decreased as the time engaged in exercise increased (e.g. adjusted ORs for activity at age 20: 1–2 h/wk. [compared to none] 0.91, 95% CI 0.65–1.29; 3–4 h/wk., 0.91, 95% CI 0.64–1.29; 5–9 h/wk., 0.84, 95% CI 0.62–1.14; 10–14 h/wk., 0.79, 95% CI 0.53–1.17; ≥15 h/wk., 0.62, 95% CI 0.42–0.91; p for trend 0.02).

However the remaining four studies examining these trends have found conflicting results, three of which measured exposure during adolescence [5, 16, 22]. Cook et al. [16] obtained information on physical activity duration during adolescence from mother-son pairs, and found conflicting evidence between sons’ responses and mothers’ responses: while information from the sons suggested that increasing time spent in sports during adolescence may be weakly associated with increasing odds of TC (e.g. adjusted ORs at age 11–13: 8–12 h/wk. [compared to <=7 h/wk], 1.09, 95% CI 0.78–1.52; 13–20 h/wk., 1.19, 95% CI 0.84–1.67; ≥21 h/wk., 1.21, 95% CI 0.82–1.78; p for trend 0.29), evidence from their mothers pointed in the opposite direction (e.g. adjusted ORs at age 11–13: 8–12 h/wk., 0.60, 95% CI 0.42–0.85; 13–20 h/wk., 0.68, 95% CI 0.49–0.95; ≥21 h/wk., 0.52, 95% CI 0.35–0.77; p for trend < 0.01).

Srivastava et al. [5] found that a higher frequency of moderate and strenuous recreational activity during teenage years was associated with greater odds of TC risk (e.g. adjusted ORs for moderate recreational activity: 1–2 times/wk. [compared to ≤3 times/mth], 1.15, 95% CI 0.54–2.44; 3–5 times/wk., 1.77, 95% CI 0.88–3.53; > 5 times/wk., 2.36, 95% CI 1.20–4.64; p for trend 0.03 [calculated based on crude data provided by authors]). Similar increasing TC risk was reported by Littman et al. [22] for increasing frequency of moderate-intensity activities during adolescence (adjusted ORs: 2- < 5 h/wk. [compared to < 2 h/wk], 1.10, 95% CI 0.70–1.60; 5- < 9 h/wk., 1.20, 95% CI 0.80–1.70; ≥9 h/wk., 1.40, 95% CI 0.90–2.10; p for trend 0.05); however the authors found no such dose-response relationship when looking at the impact of vigorous-intensity physical activity or sedentary-type activities. [22] Finally, Thune et al. [8] found no evidence that increasing intensity of recreational activity was associated with increased risk of TC development (adjusted RRs: moderate activity [compared to sedentary], 1.22, 95% CI 0.55–2.69; regular training, 1.01, 95% CI 0.41–2.49 p for trend 0.82 [calculated based on crude data provided by authors]).

### Types of recreational physical activity

Three case-control studies [6, 20, 22] investigated the association between individual types of sports and risk of TC (Table 2). All three studies examined cycling and horse-riding, while Coldman et al. [6] and Littman et al. [22] also both examined motorcycling and soccer. Forman et al. [20] had a more comprehensive exploration that also included contact sports, racquet sports, water sports, athletics, martial arts, cricket, baseball and rounders.

**Table 2:** Extracted data relating to types of recreational physical activity

Author	Study design	Exposure	Level of exposure/ comparator	Measure of relative risk (OR/RR/HR)	Reported crude OR/RR/HR (95% CI)	Reported adjusted OR/RR/HR (95% CI)	Number of exposed vs. nonexposed cases	Number of exposed vs. nonexposed controls/cohort	Adjustment for confounding
Coldman [6]	CCS	Cycling Horse-riding Motorcycling Soccer	Regular participation throughout lifetime	OR	Cycling 1.99 (1.04–3.81)		44 exposed 84 non-exposed (34% exposed)	28 exposed 100 non-exposed (22% exposed)	Controls matched for age and year of diagnosis but analysed in unmatched fashion Adjust cycling and horse-riding for cryptorchidism, juvenile onset inguinal hernia and for each other
					Horse-riding 3.31 (1.36–8.25)		25 exposed 103 non-exposed (20% exposed)	9 exposed 119 non-exposed (7% exposed)	
					Motorcycling 1.04 (0.43–2.41		16 exposed 112 non-exposed (13% exposed)	15 exposed 113 non-exposed (12% exposed)	
					Soccer 1.11 (0.50–2.50)		8 exposed 110 non-exposed (14% exposed)	16 exposed 112 non-exposed (13% exposed)	
		Cycling as a teenager for recreation or for transport to school	Regular participation as a teen		In teens Cycling for recreation 1.81 (0.88–3.75)		62 exposed 66 non-exposed (48% exposed)	49 exposed 79 non-exposed (38% exposed)	
					Cycling to school 1.79 (0.91–3.55)		40 exposed 88 non-exposed (31% exposed)	27 exposed 101 non-exposed (21% exposed)	
		Frequency of horse riding	≥1 time/mth for ≥1 year		Frequent v.s. non-frequent 2.09 (0.88–5.08)		21 exposed 107 non-exposed (16% exposed)	11 exposed 117 non-exposed (9% exposed)	
Forman [20]	CCS	Cycling Horse-riding Contact sports (football, rugby, hockey, lacrosse) Racquet sports Water sports Athletics Cricket, baseball and rounders Martial arts	Regular participation at ref. age, age 16 and age 20	OR		Cycling, horse-riding, racquet sports, cricket, baseball and rounders Unrelated to TC risk	*Information not provided*	*Information not provided*	Controls matched for age Additional adjustment for cryptorchidism, juvenile onset inguinal hernia and total exercise
						Contact sports At ref. age 0.73 (0.55–0.97) At age 20 0.80 (0.64–1.00			
						Water sports At ref. age 0.87 (0.66–1.16) At age 16 0.74 (0.58–0.96) At age 20 0.74 (0.56–0.98			
						Athletics At ref. age 0.70 (0.51–0.97			
						Martial arts At ref. age 0.67 (0.29–1.54) At age 16 0.42 (0.21–0.82) At age 20 0.52 (0.29–0.94)			
Littman [22]	CCS	Cycling Horse-riding Motor biking Mountain biking Soccer Basketball Wrestling	Regular participation during grades 7–12-yes/no	OR		Cycling 1.0 (0.7–1.3)	250 exposed 141 non-exposed (64% exposed)	658 exposed 365 non-exposed (64% exposed)	Controls matched for age and area of residence Additional adjustment for income, ethnicity, cryptorchidism. Each activity further adj. For all other activities
						Horse-riding 0.8 (0.6–1.1)	68 exposed 323 non-exposed (17% exposed)	210 exposed 813 non-exposed (21% exposed)	
						Motor biking 0.9 (0.7–1.2)	144 exposed 247 non-exposed (37% exposed)	409 exposed 614 non-exposed (40% exposed)	
						Mountain biking 0.9 (0.6–1.3)	51 exposed 263 non-exposed (16% exposed)	143 exposed 734 non-exposed (16% exposed)	
			Duration of participation during grades 7–12 in tertiles			Soccer 1–8 months 1.9 (1.2–3.1) Basketball 1–8 months 1.5 (1.0–2.2) Wrestling 6–11 months 0.4 (0.2–0.8) ≥12 months 0.8 (0.4–1.4)			

In terms of cycling, Coldman et al. [6] reported that cyclists who regularly cycled during their lifetime had almost twice the odds of developing TC than non-cyclists (age-adjusted OR: 1.98, *p* = 0.06) – although this observation was based on 44 cases and 28 controls. Two much larger studies [20, 22] reported no association between cycling as a teenager or throughout lifetime and risk of TC: for example, in a study of *n* = 391 cases and *n* = 1023 controls, Littman et al. [22] observed that those who cycled regularly during grades 7–12 were no more likely to develop TC than those who did not (adjusted OR 1.00, 95% CI 0.70–1.30).

In terms of horse-riding and risk of TC, Coldman et al. [6] observed that regular horse riding more than tripled the odds of TC (adjusted OR:3.31, 95% CI 1.36–8.25), while Littman et al. [22] observed no such evidence of an association in their larger sample (adjusted OR: 0.80, 95% CI 0.60–1.10). Forman et al. [20] stated that they found no association between horse riding and TC development, but did not report point estimates or confidence intervals for this null result. The two studies that examined the relationship between motorcycling and TC development found no evidence of association (Coldman et al. [6]: adjusted OR: 1.04, 95% CI 0.43–2.41; Littman et al. [22] adjusted OR: 0.90, 95% CI 0.70–1.20).

Of the two studies that examined soccer, Littman et al. [22] reported a reasonably strong positive association (adjusted OR: 1.90, 95% CI 1.20–3.10) while Coldman et al. reported no evidence of association [6] (adjusted OR: 1.11, 95% CI 0.50–2.50). Finally, Forman et al. [20] noted that contact sports, water sports, athletics and martial arts both during adolescence and around the ‘index’ date (i.e. around diagnosis for cases, or interview for controls) had a protective effect against conditions that are risk factors for TC (cryptorchidism and inguinal hernia-adjusted ORs ranged from 0.42 to 0.87), while racquet sports, cricket, baseball and rounders were not associated.

### Timing of physical activity

Six studies (5 case-control, 1 cohort) investigated how timing of physical activity contributed to the risk of TC by collecting physical activity data at different lifetime periods, mainly during adolescence and early adulthood (Additional file 1: Table S3). Some studies observed that the associations reported from childhood and adolescence appeared marginally stronger than those reported in adulthood (e.g. around the interview period): for example, Srivastava et al. [5] found that strenuous physical activity more than five times per week during teenage years more than doubled the risk of TC development later in life (adjusted OR: 2.58, 95% CI 1.14–5.85), but when asked the same questions regarding the two-year period prior to the interview, this association effectively disappeared (adjusted OR: 1.18, 95% CI 0.52–2.65). However, when viewed collectively the strength of the association between physical activity and TC risk does not appear to be modified substantially by the timing of exposure measurement (Fig. 2).

### Tumour histology

Three case-control studies investigated the association between physical activity and risk of TC by histology of the tumour, comparing seminomas and non-seminomas.. Again, observations were mixed: Cook et al. [16] obtained information on physical activity in adolescence from both sons and mothers, with sons’ responses suggesting a weak inverse association with seminoma (e.g. ≥21 h/wk. v.s. ≤7 h/wk. at age 11–13 adjusted OR: 1.41, 95% CI 0.81–2.45) whereas mothers’ responses suggested a strong protective effect on risk of non-seminoma (e.g. ≥21 h/wk. v.s. ≤7 h/wk. at age 11–13 adjusted OR: 0.43, 95% CI 0.27–0.68). Coupland et al. [17] noted that engaging in contact sports at age 20 and reference (or ‘index’) age may be more protective for seminoma (at age 20 adjusted OR: 0.71, 95% CI 0.54–0.93; index. Age adjusted OR: 0.52, 95% CI 0.35–0.78) than for non-seminoma/mixed (at age 20 adjusted OR: 0.91, 95% CI 0.69–1.19; ref. age adjusted OR: 0.87, 95% CI 0.64–1.19). There was no difference in risk between the two histological groups when time spent participating in exercise per week and time spent sitting down each day were examined. Lastly, Littman et al. [22] observed that moderate-intensity activity during grades 7–12 increased the risk of non-seminoma/mixed tumours (e.g. ≥9 h/wk. v.s. < 2 h/wk. adjusted OR: 2.10, 95% CI 1.10–3.90), but was not associated with seminoma (e.g. ≥9 h/wk. v.s. < 2 h/wk.: adjusted OR 1.10, 95% CI 0.70–1.80).

## Discussion

The current review pooled all available evidence regarding the relationship between physical activity among males and risk of testicular cancer, in an effort to provide clarity regarding a) whether physical activity is associated with risk of TC; b) whether a dose-response relationship exists between physical activity and risk of TC; c) whether certain types of physical activity are more strongly associated with TC than others; and d) whether exposure during certain life course periods affects TC risk more than other periods.

The studies included in this review measured and analysed physical activity exposure in a highly heterogeneous way, and even in those instances where patterns might be established by pooling results from studies with similar measures of exposure, the evidence remained inconclusive. While some studies have observed a protective effect of high levels of previous physical activity on TC risk, this observation is not consistent across studies. As shown in the forest plot, when comparing high to low levels of physical activity the magnitude of the relationship ranged considerably (Fig. 2). It is also worth noting that the magnitude of the impact of physical activity on TC risk does not correspond to the measures of exposure. For example, the risk of TC in one study that compared physical activity 1–2 times/wk. vs. none during adulthood [19] is highly comparable to that observed in a study comparing > = 15 h/week vs. none (Fig. 2) [20]. Given the heterogeneity we have observed, it would seem that there is no strong evidence that high (or moderate) levels of physical activity are associated with an increased or decreased risk of TC.

Conflicting findings were also reported for TC development and both dose-response associations and comparison of risk between individual types of sports. With respect to cycling and horse riding – two sports with potential for repetitive testicular trauma – there is not enough evidence to draw a conclusion about whether these sports are associated with higher TC risk. Perhaps the strongest evidence in this respect comes from Littman et al. [22] who observed no evidence of an association for either activity among their large sample.

### Why is there a lack of clear evidence?

#### Risk of misclassification and misclassification bias

The observational studies included in this review have several limitations that might reveal the source of the considerable heterogeneity in findings. First and foremost, one acknowledged limitation of research on physical activity and cancer risk is the complexity of assessing physical activity as an exposure due to its multifaceted nature. The lack of standard definitions of physical activity has led to the development of a wide range of subjective and objective measurement tools; most have not been tested for validity or reliability, and they do not adequately assess all parameters and timing of physical activity [4].

The use of self-report to measure physical activity is another potential limitation, and was employed in the majority of included studies (by necessity in case-control studies). Analysis shows that simultaneously measured self-reported and objective measures of physical activity are very poorly correlated; [24] in other words, the activity that adults report as having completed bears little relationship to the activity that was actually performed. It follows that retrospective estimation of physical activity exposure is likely to be highly problematic. This will be particularly true of adolescent exposure, which may have occurred many decades prior to a study. This poor recall may lead to non-differential misclassification of physical activity exposure among cases and controls, which could generate bias in risk estimates, most likely underestimating any associations. However, in studies that retrospectively assessed activity, there is also a risk of recall bias where cases (or their mothers) report activity systematically differently to controls (differential bias). This is particularly likely to occur if cases (or their mothers) believe the physical activity may have contributed to the cancer. If this is correct, we might expect to find physical activity having a greater harmful effect than is truly the case. The likelihood of misclassification of physical activity due to self-report is evident in the fact that studies assessing the (same) underlying exposure to physical activity based on reports from different sources (i.e. mothers and sons) observe completely disparate associations with TC [16]. These measurement errors collectively, and working differently in different contexts and studies, could potentially contribute to the wide variation of results found across included studies.

#### Confounding

The covariates that were included as possible confounders varied between studies. While age was commonly included as a confounder, there was variability with respect to accounting for other potential confounders. Some studies adjusted for smoking [5, 9, 18], others for history of cryptorchidism [6, 7, 16, 20–22] and others for Body Mass Index [5, 8]. Those studies that presented both crude and adjusted point estimates reported little difference between the two, suggesting that those covariates had little impact on the strength of the association between physical activity and testicular cancer. Interestingly, few studies adjusted for socioeconomic status, despite the likelihood that this measure could confound the relationship between TC risk and occupational physical activity levels in particular (both TC risk and occupation are clearly patterned by socioeconomic status). However, the study by Dosemeci et al. [18] – who found no relationship between occupational physical activity and TC risk – presented adjusted odds ratios with and without additional adjustment for socioeconomic status, and observed no difference (adjusted OR, without SES adjustment: 1.00, 95% CI 0.70–1.60; with SES adjustment: 1.00, 95% CI 0.50–1.80). The inconsistency observed with respect to the association between physical activity and TC suggests that there is unlikely to be a single source of residual confounding that might a) have significantly affected any of the studies included in the current review, or b) explain the inconsistency between published studies in terms of association between physical activity and TC development. Given the variation in study designs and measurements of physical activity, there may be particular confounders that are specific to given study types and for different life-course exposure periods; for example, we would expect different confounders for the association between occupational activity in adulthood and TC than we would expect for recreational activity in adolescence and TC.

### Biological plausibility

There are currently two pathways proposed in the literature regarding the possible biological mechanisms underlying the putative relationship between physical activity and TC, both of which relate to changes in hormone activity. The first pathway is that sporting activities that apply pressure or trauma to the male genitalia, such as horse riding, bicycling and motorcycling, are likely to cause testicular trauma or injury TC [6, 20, 22]. Severe testicular trauma or injury could lead to testicular atrophy and thereby reduced androgen synthesis [22]. This provides a possible explanation for the harmful effect of horse riding and bicycling on TC risk seen by Coldman et al. [6]; however, as we have noted other studies have not found such an association [20, 22].

The aetiological role of testicular trauma or injury in TC development remains controversial due to a) the difficulty in both defining and measuring subclinical testicular trauma or injury, b) the possibility that men with TC are more likely to recall such injury compared to men without TC (i.e. recall bias), and c) the possibility that the act of seeking clinical treatment for such injury increases the likelihood for men to be diagnosed with TC (i.e. surveillance bias).

The second proposed pathway between physical activity and TC risk is held to be mediated through changes in reproductive hormonal levels. The timing of this pathway seems plausible, since the peak incidence of TC occurs among young men within two decades of puberty – a period during which reproductive hormones are most likely to be influential. Although the specific mechanisms of how physical activity affects male hormonal levels are unclear, current research suggests that physical activity may lead to modulation in androgen levels. There is evidence that exercise has long-term effects on testosterone levels, [25, 26] while some studies have observed both an increase [27] and a decrease [28] in male’s testosterone levels shortly after exercise. In turn, it is hypothesised that androgen and gonadotrophin levels can influence TC development: high gonadotrophin levels and low testosterone levels are hypothesized to be related to increased TC risk, as ‘gonadotrophin overdrive’ in response to low testosterone levels may stimulate neoplasm progression [3]. Given hormonal regulation is related to both physical activity and TC development, and the plausibility of adolescence/early adulthood as a critical window in which exposure may affect TC risk, this hypothesis requires further examination.

### Strengths and limitations of review

The strengths of this review include a comprehensive literature search, clear and concise inclusion/exclusion criteria, the use of a PICOS statement and adherence to PRISMA guidelines, and a thorough assessment of studies for risk of bias against Newcastle-Ottawa Quality Assessment Scale.

A limitation of this review is that, due to heterogeneity in exposure measurement, when interpreting the available evidence, we grouped physical activity into high, moderate and low exposure groups regardless of how this exposure was measured (Additional file 1: Table S2). This was necessary in order to consider the evidence in as consistent a manner as possible; however, we recognise that this categorization is non-specific regarding the various aspects of physical activity (frequency, intensity, duration or type). Another limitation is the large difference in sample sizes across included studies, ranging from fewer than 50 cases [8, 23] to over 500 cases [7, 21]. This offers a possible explanation for the inconsistency seen in reported risk estimates and width of confidence intervals, with smaller sample sizes generating wider CIs and hence reflecting greater uncertainty than larger ones.

### Recommendations for future research

#### Standardised physical activity measurement

In order to adequately assess the association between physical activity and risk of TC and specifically to reduce concerns regarding the impact of bias, there is a need for more high quality studies that use integrated, validated and reliable measurements of net physical activity exposure. While some current measurement tools are better than others in this regard, it may be said that none of them truly fulfill these criteria. The commonality of the case-control study design in investigations of TC aetiology makes the absence of a valid tool for retrospectively determining physical activity particularly problematic; thus, a solution to this problem is sorely needed.

#### Exercise and changes to sex hormone production

Considering the potential etiological role of gonadotrophin and significance of early life exposure in TC development, we recommend further research in the plausibility of hormonal regulation as the underlying biological mechanism involved in the pathway between physical activity and TC development, specifically on the mechanisms of how exercise affects hormonal profiles of men and whether the influence that exercise exerts on hormonal levels varies for males in childhood, adolescence or adulthood.

#### Genetics

While there is some evidence that propensity to physical activity might be influenced by genetic factors, there is currently a paucity of genetic loci that have been robustly associated with regular physical activity [29]. Assuming that large-scale genomic studies will identify one or more genetic variants that predispose an individual to undertake high levels of physical activity, future studies (such as those driven by the Testicular Cancer Consortium, or TECAC) should permit the examination of whether loci linked to physical activity are also associated with testicular cancer. In addition, a Mendelian randomisation approach to investigating causality between this exposure and testicular cancer may be useful. This approach is not subject to biases such as misclassification bias, since the presence of the genetic variant is used as a proxy for the exposure.

## Conclusions

The current state of evidence regarding the relationship between physical activity and testicular cancer remains inconclusive. While some studies have observed a strong protective effect, others have not; and while dose-response relationships have been observed across several studies, these have been reported in both directions. Similarly conflicting results have been found in terms of the effect that individual types of activity and the timing in which activity is performed has on TC risk.

Overall, our ability to conclude whether physical activity is associated with TC risk based on current evidence is substantially influenced by the heterogeneity with which this relationship has been examined, differences in sample sizes, the likelihood of misclassification bias, the possibility of uncontrolled confounding and the potential for recall bias. However, taking these caveats into consideration, on balance we are led to suggest that there is currently no strong evidence of an association between physical activity and risk of subsequent TC.

This lack of evidence should not be interpreted as non-existence of a relationship. Our review highlights several important areas for future research, most notably on the need for an integrated, objective approach to measure physical activity, a deeper understanding of underlying biological mechanisms involved and the potential of using genetics to examine this association.

## Additional files

Additional file 1: **Table S1.** Papers included in meta-analysis of association between physical activity and testicular cancer risk, with study meta-data. **Table S2.** Extracted data relating to high vs. low physical activity. **Table S3.** Extracted data relating to high vs. low recreational physical activity at adolescence/early adulthood. **Table S4.** PICOS (Patient/Participant, Intervention, Comparator, Outcome, Study design) criteria for inclusion of studies. **Table S5.** List of excluded papers with reason for exclusion. **Table S6.** Assessment of study quality against Newcastle-Ottawa criteria for case-control studies. **Table S7.** Assessment of study quality against Newcastle-Ottawa criteria for cohort studies. (DOCX 134 kb)

## Abbreviations

TC: Testicular Cancer
TECAC: Testicular Cancer Consortium
