# Supplementary material for: Physical activity and risk of testicular cancer: a systematic review
Source: BMC Cancer. 2018 Feb 14;18:189. doi: 10.1186/s12885-018-4093-3 (PMC5813362; doi:10.1186/s12885-018-4093-3)
Supplement: Supplementary file 1 — Table S1. Papers included in meta-analysis of association between physical activity and testicular cancer risk, with study meta-data. Table S2. Extracted data relating to high vs. low physical activity. Table S3. Extracted data relating to high vs. low recreational physical activity at adolescence/early adulthood. Table S4. PICOS (Patient/Participant, Intervention, Comparator, Outcome, Study design) criteria for inclusion of studies. Table S5. List of excluded papers with reason for exclusion. Table S6. Assessment of study quality against Newcastle-Ottawa criteria for case-control studies. Table S7. Assessment of study quality against Newcastle-Ottawa criteria for cohort studies. (DOCX 134 kb) [file 12885_2018_4093_MOESM1_ESM.docx]

**Table S1: Papers included in meta-analysis of association between physical activity and testicular cancer risk, with study meta-data.**

| Author | Year of publication | Study design | Study period | Location of study | Sample size | Source of case/  outcome | Source of control/  total cohort | Exclusion criteria | Method of exposure measurement | Timing of exposure measurement | Method of outcome measurement |
| --- | --- | --- | --- | --- | --- | --- | --- | --- | --- | --- | --- |
| Brownson [1] | 1991 | CCS | 1984- 1989 | Missouri, USA | 252 cases  16895 controls | Missouri Cancer Registry  (TC registrations) | Missouri Cancer Registry  (other cancer registrations) | -Age <20 yr  -Non- European males  -Cancer of unknown primary sites  -No or non-informative job info | Occupational data gathered on admission to hospital using a standardized  protocol,  then  categorized by occupation according to the  level of physical activity (low, moderate and high) using a classification scheme | Retrospective, on admission to hospital | Clinical  diagnosis |
| Coldman [2] | 1982 | CCS | 1970- 1977 | Vancouver,  Canada | 128 cases  128 controls | Single hospital | Single hospital skin cancer or Hodgkin’s disease patients | -Non-seminoma or mixed type germ cell tumours  -Non-germ cell tumours  -Addresses of participants not known  -Deceased at the time of study | Self-report questionnaires | Retrospective, period before diagnosis and for cycling at different life periods | Clinical diagnosis |
| Cook [3] | 2008 | CCS | 2002- 2005 | USA | 499 cases  539 controls | Participants with at least one serum sample stored in Department of Defense Serum Repository (DoDSR) | Participants with at least one serum sample stored in Department of Defense Serum Repository (DoDSR) | -Age <18 and >45 yr  -Non-germ cell tumours | Computer- assisted telephone interview with structured questionnaire for each member of mother-son pair | Retrospective,  throughout childhood/adolescence (1^st^-5^th^ grades, 6^th^-8^th^ grades, 9^th^-12^th^ grades) | Clinical diagnosis |
| Coupland [4] | 1999 | CCS | 1984- 1986 | England and Wales, UK | 794 cases  794  controls | Cancer treatment centres & regional cancer registries | Age matched controls from GP registers | -Age <15 and >49 yr  -Non-germ cell tumours  -Non-European males  -Men with previous malignancy or with psychiatric conditions  -Non-resident in study area  -Cases diagnosed abroad  -Controls moved out of study area | Face to face interview  GP notes & mothers’ self-report questionnairescconfirm medical history | Retrospective, at 16 yr, 20yr and reference age (1yr before diagnosis for case and date control would have been the equivalent age) | Clinical diagnosis |
| Dosemeci [5] | 1993 | CCS | 1979- 1984 | Istanbul, Turkey | 191 cases,  2127 controls | Single hospital records | Single hospital records | -Incomplete data on occupation or smoking | Occupational titles from hospital record coded using a classification system and job-exposure matrix for occupational physical activity created based on this this classification with two physical activity indices (energy expenditure and sitting time) | Retrospective, work history after age 20 | Clinical diagnosis |
| Dusek [6] | 2008 | CCS | 2000- 2006 | Czech Republic | 356 cases  317 controls | Two Czech cancer centres | Blood donors, non-familial contacts of patients and hospital personnel | -Age <18 and >64 yr  -Non- European males  -Non-germ cell tumours  -Incomplete or incorrect data on demographic, parameters and medical history  -Men whose family history are not known | Standardised questionnaire filled in with the assistance of a physician | Retrospective, for occupational physical activity including current work and night work at least 3 yr before diagnosis/questionnaire start date, for recreational physical activity at any age, professional sport at any age and at puberty, types of sport at any age | Clinical diagnosis |
| Forman [7] | 1994 | CCS | 1984- 1987 | England and Wales, UK | 794 cases  794 controls | Treatment centres & regional cancer registries | Age matched controls from GP registers | -Age <15 and >49 yr  -Non-germ cell tumours  -Non-European males  -Men with previous malignancy or with psychiatric conditions  -Non-resident in study area  -Cases diagnosed abroad  -Controls moved out of study area | Face to face interview  GP notes & mothers’ self-report questionnairescconfirm medical history | Retrospective,  at 16 yr, 20yr & reference age (1yr before diagnosis for case and date control would have been the equivalent age) | Clinical diagnosis |
| Forman [8] | 1994 | CCS | 1984- 1987 | England and Wales, UK | 794 cases  794 controls | Treatment centres & regional cancer registries | Age matched controls from GP registers | -Age <15 and >49 yr  -Non-germ cell tumours  -Non-European males  -Men with previous malignancy or with psychiatric conditions  -Non-resident in study area  -Cases diagnosed abroad  -Controls moved out of study area | Face to face interview  GP notes & mothers’ self-report questionnaires confirm medical history | Retrospective,  at 20yr & reference age (1yr before diagnosis for case and date control would have been the equivalent age) | Clinical diagnosis |
| Gallagher [9] | 1995 | CCS | 1980- 1985 | British Columbia and Alberta, Canada | 510 cases  996 controls | Provincial cancer registries | Group 1: Subscriber list of medical insurance which enrolls nearly the total population in British Columbia Group 2: Motor vehicle license registry in Alberta | -Age <15 and >79 yr  -Non-germ cell tumours  - Non-resident of study area  - Patients’ physicians do not consent | Self-report questionnaire  Phone interview to obtain missing data | Retrospective,  for occupational physical activity >20 yr & for recreational physical activity annual frequency of participation in all sports played and duration of years played | Clinical diagnosis |
| Littman [10] | 2009 | CCS | 1999- 2006 | Western Washington, USA | 391 cases  1023 controls | Population based cancer registry | Random digit telephone dialing frequency matched by age and area of residence | -Age <18 and >44 yr  -Non-germ cell tumours  -Non-English speaking  -Non-resident in study area  -No landline telephone at home  -- Patients’ physicians do not consent | Face to face interview using structured questionnaire | Retrospective, throughout childhood/adolescence (7^th^-12^th^ grades) | Clinical diagnosis |
| Paffenbarger [11] | 1987 | Cohort | 1916- 1950 | Harvard College and University of Pennsylvania, USA | 51977 men | Death certificates | Harvard College and University of Pennsylvania alumini | Unspecified | College records and return-mail self-report questionnaires | Retrospective cohort, college records from 1916-1950 and return-mail  questionnaires in the l960s and 1970s | Death certificates |
| Srivastava [12] | 2000 | CCS | 1995- 1996 | Ontario,  Canada | 212 cases  251 controls | Ontario Cancer registry | Ontario Ministry of Finance Property Assessment database | -Age <20 and >74 yr  -Non-germ cell tumours | Self-report questionnaire  Phone interview follow-up | Retrospective,  for occupational physical activity at early 20s, early 30s and reference age (2 yr ago) & for recreational physical activity at teen, early 30s, early 50s and reference age (2 yr ago) | Clinical diagnosis |
| Thune [13] | 1994 | Cohort | 1972- 1978 | Norway | 53242 men | Norway cancer registry | Population based health screening program for cardiovascular disease in 3 counties (Oppland, Sogn and Fjordane, and Finnmark) and 2 cities (Oslo and Tromso) | -Age <19 and >50 yr  -Non-resident in study area  -Previous malignancy or diagnosed with malignancy within 1^st^ yr of enrollment | Self report questionnaire | 1 year before questionnaire | Clinical diagnosis |

**Table S2: Extracted data relating to high vs. low physical activity**

| Author | Study design | Exposure | Level of exposure/  comparator | Measure of relative risk  (OR/RR/HR) | Reported **crude** OR/RR/HR  (95% CI) | Reported **adjusted** OR/RR/HR (95% CI) | Number of exposed vs. non- exposed cases | Number of exposed vs. non- exposed controls/cohort | Adjustment for confounding |
| --- | --- | --- | --- | --- | --- | --- | --- | --- | --- |
| Brownson [1] | CCS | Occupational physical activity based on job titles | Physical activity required more  than 80 percent of the time (high activity),  20-80 percent of the time (moderate activity)  or less than 20 percent of the time  (low activity) | OR |  | Low v.s. High  2.2 (1.3-3.7) | 32 exposed  31 non-exposed  (51% exposed) | 2,426 exposed  1,019 non-exposed  (70% exposed) | Adjusted for age and smoking |
|  |  |  |  |  |  | Moderate v.s. High  1.1 (0.8-1.7) | 32 exposed  189 non-exposed  (14% exposed) | 2,426 exposed  13450 non-exposed  (15% exposed) |  |
| Dosemeci [5] | CCS | Occupational physical activity | Energy expenditure index (sedentary <8 kJ/min, moderate 8-12 kJ/min, active >12 kJ/min)  Hours spent sitting down per day (active <2 h, moderate 2-6 h, sedentary >6 h) | OR | <8 kJ/min v.s. >12 kJ/min  1.0 (0.7-1.6)  8-12 kJ/min v.s. >12 kJ/min  1.2 (0.8-1.9)  >6 h/day v.s. <2 h/day  1.0 (0.6-1.6)  2-6 h/day v.s. <2 h/day  1.0 (0.7-1.4) | <8 kJ/min v.s. >12 kJ/min  1.0 (0.5-1.8)  8-12 kJ/min v.s. >12 kJ/min  1.2 (0.8-1.8)  >6 h/day v.s. <2 h/day  0.7 (0.4-1.5)  2-6 h/day v.s. <2 h/day  0.9 (0.6-1.4) | 54 exposed  48 non-exposed  (53% exposed)  89 exposed  48 non-exposed  (65% exposed)  23 exposed  113 non-exposed  (17% exposed)  55 exposed  113 non-exposed  (33% exposed) | *Information not provided* | Adjusted for age, smoking and SES |
| Dusek [6] | CCS | Occupational physical activity and recreational physical activity | OPA  Ever had v.s. none  Night work (at least 3 yr prior to diagnosis/interview) v.s. none  RPA  Non-professional physical activity 1-2 times/wk v.s. none | OR |  | All TC  Ever had v.s. none  2.26 (1.65-3.10)  Night work v.s. none  1.48 (1.07-2.06)  1-2 times/wk v.s. none  0.49 (0.32-0.72) | 51% exposed  39% exposed  72% exposed | 32% exposed  29% exposed  85% exposed | Controls matched for age |
| Forman [14] | CCS | Recreational physical activity | Hours of exercise per week (none, 1-2 h, 3-4 h, 5-9 h, 10-14 h, ≥15 h)  Hours spent sitting down per day (0-2 h, 3-4 h, 5-6 h, 7-9 h, ≥10 h) | OR | At ref. age  ≥15 h/wk v.s. none  0.50  At ref. age  ≥10 h/day v.s. 0-2 h/day  1.59 | At ref. age  ≥15 h/wk v.s. none  0.54 (0.32-0.90)  At ref. age  ≥10 h/day v.s. 0-2 h/day  1.71 (1.08-2.72) | 28 exposed  331 non-exposed  (8% exposed)  227 exposed  52 non-exposed  (81% exposed) | 50 exposed  309 non-exposed  (14% exposed)  184 exposed  62 non-exposed  (75% exposed) | Controls matched for age  Additional adjustment for cryptorchidism and juvenile onset inguinal hernia |
| Gallagher [9] | CCS | Occupational physical activity  and recreational physical activity | Occupational activity aged 20+ of 1 year or longer, calculated to a cumulative job activity score which is divided by working years to obtain an activity score, analysed in quartiles  (level 1-lowest, level 2, level 3 and level 4- highest)  Annual frequency of participation in all sports played and duration of years played, converted to mean, annual MET weighted frequency, analysed in quartiles  (0-242-lowest, 243-441, 442-764, 765+-highest) | OR | Level 4 v.s. Level 1  0.9  765+ v.s. 0-242  0.7 | Level 4 v.s. Level 1  0.9 (0.6-1.3)  765+ v.s. 0-242  0.7 (0.5-0.9) | 87 exposed  117 non-exposed  (43% exposed)  118 exposed  160 non-exposed  (42% exposed) | 189 exposed  221 non-exposed  (46% exposed)  248 exposed  247 non-exposed  (50% exposed) | Controls matched for age  Additional adjustment for ethnicity, cryptorchidism and inguinal hernia |
| Srivastava [12] | CCS | Occupational physical activity based on job duties & recreational physical activity | Intensity of lifetime occupational activity  (sitting, light, moderate, strenuous)  Frequency of moderate and strenuous recreational activities (<1 time/mth, 1-3 times/mth, 1-2 times/wk, 3-5 times/wk, >5 times/wk) | OR |  | At ref. age  Strenuous v.s. Sitting  0.94 (0.46-1.90)  Moderate v.s. Sitting  0.98 (0.55-1.75)  Light v.s. Sitting  1.32 (0.73-2.37)  At ref. age  Moderate  >5 times/wk v.s. <1/mth  1.41 (0.61-3.29)  Strenuous  >5 times/wk v.s. <1/mth  1.18 (0.52-2.65) | 31 exposed  59 non-exposed  (34% exposed)  48 exposed  59 non-exposed  (45% exposed)  48 exposed  59 non-exposed  (45% exposed)  43 exposed  18 non-exposed  (70% exposed)  21 exposed  41 non-exposed  (34% exposed) | 37 exposed  66 non-exposed  (36% exposed)  60 exposed  66 non-exposed  (48% exposed)  42 exposed  66 non-exposed  (39% exposed)  42 exposed  23 non-exposed  (65% exposed)  23 exposed  57 non-exposed  (29% exposed) | Controls matched for age  Additional adjustment for BMI, education, smoking (pack years, years since quiting) and marital status  ORs for moderate and strenuous activity adjusted for occupational activity and vice versa. |
| Thune [15] | Cohort | Occupational physical activity based on job duties & recreational physical activity | Occupational activity intensity-Sedentary, walking, lifting and walking, heavy manual  Recreational activity intensity-Sedentary, moderately active (RPA at least 4 h/wk), regular training (for fitness at least 4 h/wk or for competition several times/wk) | RR |  | Heavy manual v.s. Sedentary  1.95 (0.86-4.41)  Lifting and walking v.s. Sedentary  1.38 (0.63-3.01)  Walking v.s. Sedentary  0.60 (0.23-1.57)  Regular training v.s. Sedentary  1.01 (0.41-2.49)  Moderately active v.s. Sedentary 1.22 (0.55-2.69) | 13 exposed  13 non-exposed  (50% exposed)  13 exposed  13 non-exposed  (50% exposed)  6 exposed  13 non-exposed  (32% exposed)  12 exposed  8 non-exposed  (60% exposed)  26 exposed  8 non-exposed  (76% exposed) | 130,126 exposed  309,845 non-exposed  (30% exposed)  189,190 exposed  309,845 non-exposed (38% exposed)  225,925 exposed  309,845 non-exposed (42% exposed)  217,200 exposed  170,718 non-exposed  (56% exposed)  471,125 exposed  170,718 non-exposed  (70% exposed) | Adjusted for age, geographic region and BMI |

**Table S3: Extracted data relating to high vs. low recreational physical activity at adolescence/early adulthood**

| Author | Study design | Exposure | Level of exposure/  comparator | Measure of relative risk  (OR/RR/HR) | Reported **crude** OR/RR/HR  (95% CI) | Reported **adjusted** OR/RR/HR (95% CI) | Number of exposed vs. non- exposed cases | Number of exposed vs. non- exposed controls/cohort | Adjustment for confounding |
| --- | --- | --- | --- | --- | --- | --- | --- | --- | --- |
| Cook [3] | CCS | Exercise time during 1-5^th^ grades, 6-8^th^ grades and 9-12^th^ grades | Hours of sports or vigorous physical activity per week reported by sons and mothers in 3 time periods (≤5 h, 6-10 h, 11-15 h and ≥16 h for 1^st^-5^th^ grades, ≤7 h, 8-12 h, 13-20 h and ≥21 h for 6^th^-8^th^ grades, ≤8 h, 9-15 h, 16-20 h and ≥21 h for 9^th^-12^th^ grades) | OR |  | Sons:  1^st^-5^th^ grades ≥16 h/wk v.s. ≤5 h/wk  1.32 (0.93-1.85)  6^th^-8^th^ grades ≥21 h/wk v.s. ≤7 h/wk  1.21 (0.82-1.78)  9^th^-12^th^ grades  ≥21 h/wk v.s. ≤8 h/wk  1.07 (0.74-1.55)  Mothers:  1^st^-5^th^ grades ≥16 h/wk v.s. ≤5 h/wk  0.56 (0.39-0.80)  6^th^-8^th^ grades ≥21 h/wk v.s. ≤7 h/wk  0.52 (0.35-0.77)  9^th^-12^th^ grades  ≥21 h/wk v.s. ≤8 h/wk  0.60 (0.42-0.87) | 120 exposed  146 non-exposed  (45% exposed)  137 exposed  120 non-exposed  (53% exposed)  113 exposed  113 non-exposed  (50% exposed)  100 exposed  159 non-exposed  (39% exposed)  73 exposed  150 non-exposed  (33% exposed)  90 exposed  152 non-exposed  (37% exposed) | 112 exposed  178 non-exposed  (39% exposed)  90 exposed  148 non-exposed  (38% exposed)  114 exposed  129 non-exposed  (47% exposed)  134 exposed  124 non-exposed  (52% exposed)  105 exposed  115 non-exposed  (48% exposed)  126 exposed  128 non-exposed  (50% exposed) | Controls matched for age, ethnicity and serum sample date  Additional adjustment for cryptorchidism and family history of testicular cancer |
| Forman [14] | CCS | Exercise and sitting time at age 20 | Hours of exercise per week (none, 1-2 h, 3-4 h, 5-9 h, 10-14 h, ≥15 h)  Hours spent sitting down per day (0-2 h, 3-4h, 5-6 h, 7-9 h, ≥10 h) | OR | At age 20  ≥15 h/wk v.s. none  0.65  At age 20  ≥10 h/day v.s. 0-2 h/day  1.25 | At age 20  ≥15 h/wk v.s. none  0.62 (0.42-0.91)  At age 20  ≥10 h/day v.s. 0-2 h/day  1.35 (0.88-2.06) | 65 exposed  248 non-exposed  (21% exposed)  117 exposed  114 non-exposed  (51% exposed) | 86 exposed  217 non-exposed  (28% exposed)  91 exposed  104 non-exposed  (47% exposed) | Controls matched for age  Additional adjustment for cryptorchidism and juvenile onset inguinal hernia |
| Littman [10] | CCS | Exercise time during 7^th^-12^th^ grades | Hours of sedentary, moderate and vigorous intensity activities per week  (<7 h, 7-13 h, 14-20 h and ≥21 h for sedentary, <2 h, 2-4 h, 5-8 h and ≥9 h for moderate, <5 h, 5-9 h, 10-13 h and ≥14 h for vigorous)  Duration of competitive sports in months (none, 1-23 mths, 24-47 mths, ≥48 mths) | OR |  | Sedentary  ≥21 h/wk v.s. <7 h/wk  1.3 (0.8-2.0)  Moderate  ≥9 h/wk v.s. <2 h/wk  1.4 (0.9-2.1)  Vigorous  ≥14 h/wk v.s. <5 h/wk  1.0 (0.7-1.5)  ≥48 mths v.s. none  1.2 (0.8-1.7) | 131 exposed  39 non-exposed  (77% exposed)  122 exposed  54 non-exposed  (69% exposed)  122 exposed  85 non-exposed  (59% exposed)  127 exposed  64 non-exposed  (66% exposed) | 316 exposed  131 non-exposed  (71% exposed)  263 exposed  164 non-exposed  (62% exposed)  294 exposed  218 non-exposed  (57% exposed)  362 exposed  179 non-exposed  (67% exposed) | Controls matched for age and area of residence  Additional adjustment for income, ethnicity, cryptorchidism and other types of physical and sedentary activities |
| Paffenbarger [11] | Cohort | Sports participation during college | Participation in sports > 5 h/wk compared to < 5 h/wk | RR |  | >5 h/wk v.s. <5 h/wk  1.20, 0.59-2.44* | 25 exposed  45 non-exposed  (36% exposed) | *Information not provided* | Adjustment for age, birth year and sex |
| Srivastava [12] | CCS | Occupational physical activity based on job duties & recreational physical activity in teens and at early 20s | Intensity of lifetime occupational activity  (sitting, light, moderate, strenuous)  Frequency of moderate and strenuous recreational activities (<1 time/mth, 1-3 times/mth, 1-2 times/wk, 3-5 times/wk, >5 times/wk) | OR |  | At early 20s  Strenuous v.s. Sitting  1.67 (0.92-3.00)  Moderate v.s. Sitting  1.85 (1.05-3.26)  Light v.s. Sitting  1.30 (0.71-2.39)    In teens  Moderate  >5 times/wk v.s. ≤3 times /mth  2.36 (1.20-4.64)  Strenuous  >5 times/wk v.s. <1/mth  2.58 (1.14-5.85) | 61 exposed  39 non-exposed  (61% exposed)  65 exposed  39 non-exposed  (63% exposed)  41 exposed  39 non-exposed  (51% exposed)  94 exposed  18 non-exposed  (84% exposed)  82 exposed  11 non-exposed  (88% exposed) | 70 exposed  59 non-exposed  (54% exposed)  63 exposed  59 non-exposed  (52% exposed)  50 exposed  59 non-exposed  (46% exposed)  79 exposed  35 non-exposed  (69% exposed)  81 exposed  26 non-exposed  (76% exposed) | Controls matched for age  Additional adjustment for BMI, education, smoking (pack years, years since quiting) and marital status  ORs for moderate and strenuous activity adjusted for occupational activity and vice versa. |

*Confidence intervals not provided by authors. We have calculated these using point estimate and p-value data.[16]

**Table S4: PICOS (Patient/Participant, Intervention, Comparator, Outcome, Study design) criteria for inclusion of studies.**

| **PICOS Element** | **Inclusion Criteria** | **Exclusion Criteria** |
| --- | --- | --- |
| ***P****articipant* | Males | Animal studies |
| ***I****ntervention*  *(i)* | Intensity- moderate-strenuous |  |
| *(ii)* | Frequency- e.g. >5 days/wk |  |
| *(iii)* | Duration- e.g. 1h/day |  |
| ***C****omparator*  *(i)* | Intensity- sedentary-light |  |
| *(ii)* | Frequency- e.g. < 5 days/wk |  |
| *(iii)* | Duration- e.g. <1h/day |  |
| ***O****utcome*  *(i)* | Any testicular cancer |  |
| *(ii)* | Seminoma | Non-seminoma |
| *(iii)* | Non-seminoma | Seminoma |
| ***S****tudy design* | No limit placed on initial search criteria | Studies that do not report association between exposure and outcome |

**Table S5: List of excluded papers with reason for exclusion.**

| **Author** | **Year** | **Title** | **Reason for exclusion** |
| --- | --- | --- | --- |
| Asplund, C. | 2007 | Genitourinary problems in bicyclists | No primary data |
| Cao, B. | 2003 | Physical activity and cancer risk | Review manuscript (no primary data) |
| Friedenreich C. | 2001 | Physical activity and cancer prevention: From observational to intervention research | Review manuscript (no primary data) |
| Friedenreich, et al. | 2010 | State of the epidemiological evidence on physical activity and cancer prevention | Review manuscript (no primary data) |
| Garner, et al. | 2008 | Testicular cancer and hormonally active agents | No assessment of physical activity exposure |
| Haughey, et al. | 1989 | The epidemiology of testicular cancer in Upstate New York | No assessment of physical activity exposure |
| Langner, et al. | 2010 | Case-control study of male germ cell tumors nested in a cohort of car-manufacturing workers: Findings from occupational history | Assessment of occupational exposure does not specifically address physical activity |
| Leibovitch and Mor | 2005 | The vicious cycling: bicycling related urogenital disorders | Review manuscript (no primary data) |
| Leitzmann M. F. | 2011 | Physical activity and genitourinary cancer prevention | Book (no primary data) |
| Sommer, et al. | 2004 | Lifestyle issues and genitourinary tumours | Review manuscript (no primary data) |
| Southorn, T. | 2002 | Great balls of fire and the vicious cycle: A study of the effects of cycling on male fertility | Essay (no primary data) |
| Thorsen, et al. | 2003 | The level of physical activity in long-term survivors of testicular cancer | Does not assess etiological association between physical activity and testicular cancer |
| Thune and Furberg | 2001 | Physical activity and cancer risk: dose-response and cancer, all sites and site-specific | Review manuscript (no primary data) |
| Wolin, et al. | 2012 | Physical activity and urologic cancers | Review manuscript (no primary data) |

**Table S6: Assessment of study quality against Newcastle-Ottawa criteria for case-control studies.**

| Author | Year | Adequacy of case definition | Representativeness of cases | Selection of controls | Definition of controls | Comparability of cases and controls | Ascertainment of exposure | Same ascertainment for cases and controls | Non-response rate |
| --- | --- | --- | --- | --- | --- | --- | --- | --- | --- |
| Brownson [1] | 1991 | Yes, with record linkage | Consecutive or obviously representative series of cases | Hospital controls^a^ | No history of disease | Cases and controls comparable  (study controls for men’s age and other factors) | Medical records | Yes | No designation^b^ |
| Coldman [2] | 1982 | Yes, with independent validation | Consecutive or obviously representative series of cases | Hospital controls | No history of disease | Cases and controls comparable  (study controls for men’s age and other factors) | Written self-report | Yes | Same rate for both groups (response rate: 1^st^ questionnaire- cases 81%/ controls 80%, 2^nd^ questionnaire- cases 89%/ controls 88%) |
| Cook [3] | 2008 | Yes, with independent validation | Consecutive or obviously representative series of cases | Community controls^c^ | No history of disease | Cases and controls comparable  (study controls for men’s age and other factors) | Interview not blinded to case/control status | Yes | Rate different (response rate: cases 65%/ controls 58%)  Mothers response rate (94%) |
| Coupland [4] | 1999 | Same as Forman (8) (additional analysis) |  |  |  |  |  |  |  |
| Dosemeci [5] | 1993 | Yes, with independent validation | Consecutive or obviously representative series of cases | Hospital controls | No history of disease | Cases and controls comparable  (study controls for men’s age and other factors) | Medical records | Yes | No designation^d^ |
| Dusek [6] | 2008 | Yes, with independent validation | Consecutive or obviously representative series of cases | Community controls | No history of disease | Cases and controls comparable  (study controls for men’s age and other factors) | Written self-report, with assistance of physician | Yes | No designation |
| Forman [7] | 1994 | Same as below |  |  |  |  |  |  |  |
| Forman [14] | 1994 | Yes, with independent validation | Consecutive or obviously representative series of cases | Community controls | No history of disease | Cases and controls comparable  (study controls for men’s age and other factors) | Interview not blinded to case/control status | Yes | Rate different (response rate: cases 92%/ controls 77%) |
| Gallagher [9] | 1995 | Yes, with independent validation | Consecutive or obviously representative series of cases | Community controls | No history of disease | Cases and controls comparable  (study controls for men’s age and other factors) | Written self-report | Yes | Rate different (response rate: cases 80%/ controls 68% in total with gp.1: 69% and gp.2: 67%) |
| Littman [10] | 2009 | Yes, with record linkage | Consecutive or obviously representative series of cases | Community controls | No history of disease | Cases and controls comparable  (study controls for men’s age and other factors) | Interview not blinded to case/control status | Yes | Rate different (response rate: cases 68%/ controls 43%) |
| Srivastava [12] | 2000 | Yes, with record linkage | Consecutive or obviously representative series of cases | Community controls | No history of disease | Cases and controls comparable  (study controls for men’s age and other factors) | Written self-report | Yes | Rate different (response rate: cases 76%/ controls 64%) |

^a^Missouri Cancer Registry recorded data on incident cancer cases from public and private hospitals since 1972. All other cancer registrations excluding testicular cancer were used as control group. We have therefore assigned them as hospital controls.

^b^No response rate offered, but authors stated “Analysis was limited to 17,147 subjects from the Registry who had codable occupational information. The remaining subjects had no occupational information or non-informative job titles such as "re- tired." ”

^c^Study participants were from US Servicemen’s Testicular Tumor Environmental and Endocrine Determinants study (STEED), which enrolled servicemen between April 2002 and January 2005 who had at least one serum sample stored in the Department of Defense Serum Repos- itory (DoDSR, Silver Spring, Maryland, USA) and were between 18-45 years of age.

^d^No response rate offered, but authors stated “Subjects with incomplete information on occupation or smoking were removed from the study.”

**Table S7: Assessment of study quality against Newcastle-Ottawa criteria for cohort studies.**

| Author | Year | Representativeness of exposed cohort | Selection of non-exposed cohort | Ascertainment of exposure | Demonstrated outcome was not present at start of study | Comparability of exposed and non-exposed | Assessment of outcome | Sufficient follow-up length | Loss to follow-up |
| --- | --- | --- | --- | --- | --- | --- | --- | --- | --- |
| Paffenbarger [11] | 1987 | Selected group of users (college alumni) | Drawn from the same community as the exposed cohort | Secure record (college entrance physical exams records) and written self-report | Yes | Cohorts comparable  (study controls for men’s age) | Record linkage | Yes | *Information not provided* |
| Thune [15] | 1994 | Somewhat representative of the average men in the community | Drawn from the same community as the exposed cohort | Written self-report | Yes | Cohorts comparable  (study controls for men’s age and other factors) | Record linkage | Yes | 10% mortality during follow-up, unlikely to cause bias |
